# Rapid and sensitive hormonal profiling of complex plant samples by liquid chromatography coupled to electrospray ionization tandem mass spectrometry

**DOI:** 10.1186/1746-4811-7-37

**Published:** 2011-11-18

**Authors:** Maren Müller, Sergi Munné-Bosch

**Affiliations:** 1 Departament de Biologia Vegetal, Facultat de Biologia, Universitat de Barcelona, Avinguda Diagonal, 645, E-08028 Barcelona, Spain

**Keywords:** UPLC/ESI-MS/MS, Phytohormones, Auxins, Abscisic acid, Cytokinins, Gibberellins, Salicylic acid, Jasmonic acid, 1-amino-cyclopropane-1-carboxyic acid, *Rosmarinus officinalis*

## Abstract

**Background:**

Plant hormones play a pivotal role in several physiological processes during a plant's life cycle, from germination to senescence, and the determination of endogenous concentrations of hormones is essential to elucidate the role of a particular hormone in any physiological process. Availability of a sensitive and rapid method to quantify multiple classes of hormones simultaneously will greatly facilitate the investigation of signaling networks in controlling specific developmental pathways and physiological responses. Due to the presence of hormones at very low concentrations in plant tissues (10^-9 ^M to 10^-6 ^M) and their different chemistries, the development of a high-throughput and comprehensive method for the determination of hormones is challenging.

**Results:**

The present work reports a rapid, specific and sensitive method using ultrahigh-performance liquid chromatography coupled to electrospray ionization tandem spectrometry (UPLC/ESI-MS/MS) to analyze quantitatively the major hormones found in plant tissues within six minutes, including auxins, cytokinins, gibberellins, abscisic acid, 1-amino-cyclopropane-1-carboxyic acid (the ethylene precursor), jasmonic acid and salicylic acid. Sample preparation, extraction procedures and UPLC-MS/MS conditions were optimized for the determination of all plant hormones and are summarized in a schematic extraction diagram for the analysis of small amounts of plant material without time-consuming additional steps such as purification, sample drying or re-suspension.

**Conclusions:**

This new method is applicable to the analysis of dynamic changes in endogenous concentrations of hormones to study plant developmental processes or plant responses to biotic and abiotic stresses in complex tissues. An example is shown in which a hormone profiling is obtained from leaves of plants exposed to salt stress in the aromatic plant, *Rosmarinus officinalis*.

## Background

Hormones play a pivotal role in most physiological processes in plants. These structurally diverse compounds that act usually at nanomolar levels include five groups of the so-called "classic" hormones, comprising auxins, cytokinins, gibberellins (GA), abscisic acid (ABA) and ethylene, and several other plant growth regulators, including jasmonates, salicylates, brassinosteroids, polyamines or the very recently discovered strigolactones, which fit several of the criteria to be considered hormones [1-3]. Furthermore, the list of plant hormones is expected to increase due to a better understanding of plant growth and development and stress responses, and the use of technological advances in analytical methods.

Recent studies support the contention that hormone actions build a signaling network and mutually regulate several signaling and metabolic systems, such as auxins and GAs in growth regulation [4], CKs, auxins, ABA and strigolactones in apical dominance [2,5], auxins and brassinosteroids in cell expansion [6,7], ethylene and cytokinins in the inhibition of root and hypocotyl elongation [8], ethylene, ABA and GAs in some plant stress responses [9,10], or SA, JA and auxin in plant responses to pathogens [11,12] to name just a few of the reported hormonal interactions. Therefore, focusing on a single endogenous plant hormone to evaluate hormone-regulated physiological or developmental biological problems is not sufficient anymore [13].

In order to understand better the network regulation of hormone action influencing plant growth and development as well as the distribution of several hormones at the organ, cellular and sub-cellular levels, an ideal analytical method should provide a measure of multiple hormone concentrations (hormonal profiling) from a single experimental sample. Therefore several methods for the simultaneous quantification of multiple plant hormones using mass spectrometry with multiple reaction monitoring (MRM) have been developed recently. It has been reported a multiplex gas chromatography-tandem mass spectrometry (GC-MS/MS) technique for the simultaneous analysis of SA, JA, IAA, ABA and OPDA in *Arabidopsis thaliana *[13]. However, GC-MS is limited to volatile compounds and as a result it is necessary to purify and derivatize hormones prior to analysis. Another potential downside in GC-MS procedures apart from the purification and derivatization is the use of high temperatures, which can degrade thermal labile compounds [14].

An alternative to GC-MS is liquid chromatography coupled to mass spectrometry (LC-MS). A high performance liquid chromatography-electrospray ionization tandem mass spectrometry (HPLC/ESI-MS/MS) method for the simultaneous analysis of 15 plant hormones and metabolites from four different hormone classes (auxins, cytokinins, GAs and ABA) has been reported to analyze hormone regulation of thermodormancy of lettuce seeds [15]. Also, a HPLC/ESI-MS/MS method to analyze seven major classes of plant hormones including auxins, cytokinins, GAs, ABA, jasmonates, brassinosteriods and SA in *Arabidopsis thaliana *has been developed [1] . Furthermore, an ultrahigh-performance liquid chromatography electrospray ionization tandem mass spectrometry (UPLC/ESI-MS/MS) technique to analyze cytokinins, auxins, ABA and GAs in rice has been described [16] . To improve the detection limit of the negatively charged compounds they derivatized auxin, ABA and GAs with bromocholin and analyzed all compounds in the positive ion mode. However, at present this method is limited and cannot target other plant hormones such as JA and SA.

Plant hormones are structurally diverse compounds with diverse physiochemical properties. The question as to whether all plant hormones can be extracted equally well has not yet been answered. The choice of extraction methods depends not only on the target analysts but also on the matrix of the analyzed tissues. The requirements on the extraction method increase with the complexity of the sample matrix. In the literature diverse extraction solvents such as methanol, methanol-water mixtures, isopropanol, or isopropanol-water mixtures have been used with one or two extraction steps [14,15,17-19]. In addition, time-consuming multiple steps of sample preparation procedures, including sample purification, drying of sample under N_2 _and re-suspension of the residues have been reported for plant hormone extraction [14,20] which may increase the risk of hormone loss. However, the application of internal standards can provide corrections for hormone loss during sample preparation and chromatographic separation.

Here we developed a new method which allows to analyze dynamic changes in endogenous concentrations of major plant hormones and to study plant development processes or plant responses to biotic and abiotic stresses in complex sample matrices. An example is shown in which rosemary (*Rosmarinus officinalis*), an aromatic Mediterranean perennial shrub rich in secondary metabolites and epicuticular waxes, was exposed to salt stress. Soil salinity is one of the most serious environmental threats for plant survival and affects many undesirable changes in plants such as hyperionic and hyperosmotic effects, increase in reactive oxygen species and metabolic toxicity. These changes lead to growth reduction, changes in biomass allocation and phenology, leaf senescence, and finally to plant death [21-23]. It has been shown that senescence induced by salinity follows at least in part similar physiological events as drought-induced senescence [24]. Plant hormones such as ABA, ethylene and cytokinins are involved in different plant strategies to overcome the damaging effects of salinity, however, the complex hormonal response is only partly known [25,26]. The present work reports a sensitive and rapid method to quantify 17 plant hormones from seven plant classes including auxins, cytokinins, GAs, ABA, ACC (the ethylene precursor), SA and JA in complex tissues using ultra-performance liquid chromatography mass spectrometry (UPLC/ESI-MS/MS) with multiple reaction monitoring (MRM). This method allows obtaining a hormonal profiling in 6 min. Sample preparation, extraction procedures and UPLC-MS/MS conditions were optimized.

## Results and discussion

Of the 17 endogenous plant hormones investigated, Z, DHZ, 2-IP, IAA, ABA, JA, SA, ACC, GA_4_, GA_9_, GA_24 _were detected in rosemary leaves, whereas ZR, DHZR, IPA, GA_1_, GA_19_, and GA_20 _concentrations were under the limit of detection. However, the internal standards d_4_-SA, d_6_-ABA, d_5_-JA, d_5_-IAA, d_2_-GA_1_, d_2_-GA_4_, d_2_-GA_9_, d_2_-GA_19_, d_2_-GA_20_, d_2_-GA_24_, d_4_-ACC, d_6_-2iP, d_6_-IPA, d_5_-Z and d_5_-ZR were detected in all rosemary leaf extracts (d_5_-Z and d_5_-ZR were used as internal standards for Z, DHZ and ZR, DHZR).

### Extraction solvents

The extraction of plant hormones will critically determine the quality of the results obtained. Therefore the choice of the extraction solvent is very important, however, it is also challenging by the structurally diversity of plant hormones. Previously reported methods for plant hormone extraction used predominately methanol, methanol mixtures or isopropanol. Four classes of plant hormones including auxins, cytokinins, ABA and gibberellins were extracted using isopropanol:glacial acetic acid (99:1; v/v) [15]. Methanol:water:acetic acid (10:79:1) was used to extract ABA, SA and JA [27]. In other studies methanol:water:formic acid (75:20.5) was used to extract cytokinins, IAA and ABA [17,19]. We tested methanol:glacial acetic acid, 99:1 (v/v), isopropanol:glacial acetic acid, 99:1 (v/v) and different methanol:isopropanol:glacial acetic acid mixtures, 80:19:1; 60:39:1; 40:59:1; 20:79:1 (v/v/v). Thirty-five 100 mg samples of frozen rosemary leaves were extracted with 7 different extraction solvents including 5 replicates after incorporation of deuterated labeled plant hormones as internal standards.

**Figure 1:**
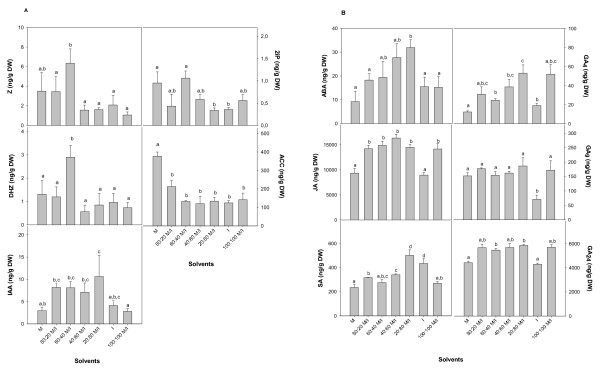
**Effects of solvents on hormone extraction**. Concentration of endogenous plant hormones (A) Z, DHZ, 2iP, IAA, ACC, and (B) ABA, JA, SA, GA_4_, GA_9_, GA_24 _detected in rosemary leaves using the following extraction solvents: M = methanol: glacial acetic acid, 99:1 (v/v); 80:20 M/I = methanol: isopropanol: glacial acetic acid 80:19:1 (v/v/v); 60:40 M/I = methanol: isopropanol: glacial acetic acid 60:39:1 (v/v/v); 40:60 M/I = methanol: isopropanol: glacial acetic acid 40:59:1 (v/v/v); 20:80 M/I = methanol: isopropanol: glacial acetic acid 20:79:1 (v/v/v); I = isopropanol: glacial acetic acid, 99:1 (v/v); 100:100 M/I = leaves were extracted first with methanol: glacial acetic acid, 99:1 (v/v) in two extraction steps and then with I = isopropanol: glacial acetic acid, 99:1 (v/v) in two extraction steps.

Due to the structurally diversity of the 17 plant hormones a solvent alone was not able to extract all plant hormones equally well (Figure 1). Whereas ABA, SA, JA, GAs and IAA showed higher yields using solvents with higher concentrations of isopropanol than methanol, opposite results were found for cytokinins and ACC. In general it could be observed that methanol:isopropanol mixtures are favorable to extract plant hormones compared to 100% methanol or 100% isopropanol except for ACC. The results suggested that the choice of the extraction solvent depends on which plant hormones are of more interest to investigate. For the analysis of ABA, SA, JA, IAA and GAs methanol:isopropanol:glacial acetic acid, 20:79:1 (v/v/v) is preferred, for cytokinins methanol:isopropanol:glacial acetic acid, 60:39:1 (v/v/v) and for ACC methanol:glacial acetic acid, 99:1 (v/v). Therefore the results indicate that plant hormones including amines (cytokinins and ACC) were dissolved preferentially in the more polar solvent whereas plant hormones including a carboxyl group (ABA, JA, SA, IAA, GAs) were dissolved in the less polar solvent. For the following experiments plant material was analyzed using the less polar extraction solvent methanol:isopropanol:glacial acetic acid, 20:79:1 (v/v/v) which is favorable to analyze ABA, SA, JA, GAs, and IAA. Recoveries about > 80% were found for ABA, SA, GA_4_, GA_9_, IAA; 77% for d_2_-GA_24_; between 62 and 50% for JA, 2iP, ACC; and 35% for Z.

### Fresh or dried leaf material

Little is known whether freeze drying (compared to fresh plant material) adversely affects plant hormone contents. A 25% decrease was observed for SA and JA yields from freeze dried compared to fresh leaf material of *Arabidopsis *[27]. A decrease of 50% in SA but no change in JA levels of freeze dried material from cucumber compared to those from the equivalent amount of fresh tissue was measured [20]. Here plant hormone contents from fresh frozen and freeze dried leaf material of rosemary were compared. Leaves were collected and immediately frozen in liquid nitrogen. Ten fresh weight (FW) and ten freeze dried samples (DW) were then extracted after the addition of internal standards. Additional File 1 shows no significant differences in plant hormone contents comparing fresh frozen and freeze dried plant material, except for GA_9_, which showed significant higher contents in fresh samples.

### Extraction steps

Undoubtedly, the requirements on the extraction methods increase with the complexity of the sample matrix. Rosemary leaves represent a complex matrix including essential oils, tannins, flavonoids, diterpenes, saponins, epicuticular waxes and resin. Five 100 mg samples (fresh weight) were extracted five times after including internal standards. Each supernatant was immediately dried under nitrogen stream, re-suspended and injected to LC-MS. Additional File 2 shows clear differences regarding the necessary extraction steps for endogenous plant hormones. DHZ was only detectable in the first three extractions; 2iP, JA, and GA_9 _in four extractions; and Z, IAA, ACC, ABA, SA, GA_4 _and GA_24 _in five extractions.

### Extract drying

**Figure 2:**
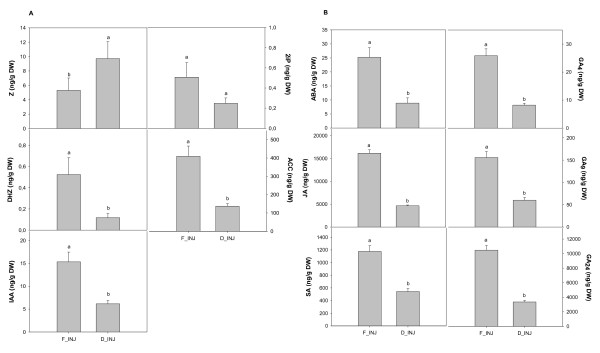
**Effects of drying and re-suspension**. Plant extract directly injected into the UPLC/ESI-MS/MS (F_INJ) compared to dried under nitrogen stream and re-suspended (D_INJ). Concentration of endogenous plant hormones (A) Z, DHZ, 2iP, IAA, ACC, and (B) ABA, JA, SA, GA_4_, GA_9_, GA_24 _found in rosemary leaves.

The concentration of sample extract by drying of samples under N_2 _and re-suspension of the residues is widespread in the literature [14,15]. However, each manipulation runs the risk of plant hormone loss apart from being time consuming. Twenty 25-50 mg samples (FW) were extracted after the addition of internal standards with 200 μl of solvent extract (methanol: isopropanol: glacial acetic acid, 20:79:1, v/v) using ultra sonication. After centrifugation (10,000 rpm for 15 min at 4°C), the supernatant was collected and the pellet was re-extracted twice with 100 μl of extraction solvent. The supernatants were combined and divided. One half was immediately injected and the other one was completely dried under nitrogen stream and re-suspended before being injected. For ABA, ACC, JA, DHZ, IAA, GA_4_, GA_9_, GA_24 _and for 2iP and SA a significant loss about 70% and 50%, respectively, could be observed for the dried extract compared to the immediately injected extract (Figure 2). Only Z showed higher levels for the dried and re-suspended extract. The high loss of plant hormones during the drying process indicates that sample extractions should immediately be injected, however, sample weight and volume of the extraction solvent must be adjusted.

### Quality parameters

In the optimum LC-MS/MS conditions calibration curves were created using solutions containing varying amounts of each unlabeled analyte compound and a known fixed amount of deuterium labeled internal standard. The obtained calibration curves showed linearity of correlation coefficients (R^2^) in the concentration range selected between 0.996 and 0.999 for the different analysts.

To test the reproducibility of the developed method sample extracts were repeatedly injected (five times). The results show good reproducibility of elution times (relative standard deviations, RSDs, < 0.73) and peak areas (RSDs, < 5.07) (Table 1) for each compound.

**Table 1:** Reproducibility of the developed LC/ESI-MS/MS method.

Analyte/IS	RRT^a^ RSD (%)	RPA^b^ RSD (%)
**Z/d_5_-Z**	0.51	4.11
**IAA/d_5_-IAA**	0.38	3.45
**ABA/d_6_-ABA**	0.20	4.36
**JA/d_5_-JA**	0.21	0.45
**SA/d_4_-SA**	0.21	0.47
**ACC/d_4_-ACC**	0.73	2.78
**GA_4_/d_2_-GA_4_**	0.20	1.72
**GA_9_/d_2_-GA_9_**	0.00	4.52
**GA_24_/d_2_-GA_24_**	0.19	1.11

^a ^Relative retention time

^b ^Relative peak area

Sensitivity parameters are listed in Table 2 where the limit of detection (LOD) and limit of quantification (LOQ) based on a signal-to-noise ratio of 3:1 and 10:1, respectively, were calculated through the standard addition curves. LOD for the different plant hormones ranged from 0.07 ng g^-1 ^for DHZ to 12.1 ng g^-1 ^for GA_20_, and LOQ ranged from 0.24 ng g^-1 ^for DHZ to 40.33 ng g^-1 ^for GA_20 _in fresh rosemary leaves. To achieve a fully quantitative determination of plant hormones in plant tissues the necessary plant material has also to be taken into account. The minimum detectable amounts in samples analyzed were calculated regarding the limit of detection for each detectable plant hormone. The data shows that less than 0.1 mg (FW) of leaf tissue is sufficient to determine ACC, SA, JA, GA_24_, while the analysis of Z, ABA, IAA, GA_4 _and GA_9 _require amounts between 3 and 9 mg (FW) and DHZ and 2iP amounts between 10 and 25 mg (FW) (Table 3). The capacity of the extraction method to analyze different amounts of leaf tissue was also tested. Twenty-five rosemary samples between 20 and 200 mg (FW) were extracted after the addition of internal deuterium labeled plant hormones. Additional File 3 shows clearly that a linear relationship exists for all detected compounds of interest over the whole range of sample sizes (R^2 ^values of 0.981 to 0.999 for all detected plant hormones).

**Table 2:** LOD and LOQ values.

Analyte	LOD^a^ (ng/g DW)	LOQ^b^ (ng/g DW)
**Z**	0.09	0.29
**ZR**	4.20	13.99
**DHZ**	0.07	0.24
**DHZR**	0.21	0.69
**2iP**	0.16	0.52
**IPA**	1.54	5.14
**IAA**	0.48	1.59
**ABA**	0.31	1.04
**JA**	1.24	4.13
**SA**	0.21	0.70
**ACC**	0.23	0.78
**GA_1_**	4.30	14.35
**GA_4_**	0.23	0.78
**GA_9_**	1.42	4.73
**GA_19_**	0.15	0.51
**GA_20_**	12.10	40.33
**GA_24_**	5.29	17.63

^a ^Limit of detection (S/N = 3)

^b ^Limit of quantification (S/N = 10)

**Table 3:** Requirements of plant material for the UPLC-MS/MS analysis of endogenous plant hormones in *Rosmarinus officinalis *plants.

Analyte	Minimum tissue requirement mg (FW)
**Z**	4.54
**DHZ**	24.38
**2iP**	24.52
**IAA**	8.62
**ABA**	2.61
**JA**	0.02
**SA**	0.03
**ACC**	0.09
**GA_4_**	1.77
**GA_9_**	1.27
**GA_24_**	0.09

### Sample stability

It is quite important to develop a method that is not only simple, sensitive and rapid, but also a high throughput screening is desirable. To test the stability, analyzed sample extracts remained in the autosampler (4°C) for 48 h and were then re-injected. Table 4 summarizes the ratio of plant hormones to internal standards at 0 and 48 h. No significant degradation of samples was found over 48 h (relative retention time, RSD < 1.35 and relative peak area, RDS < 9.47), indicating that this method allows preparing and screening about 450 samples in 2 days.

**Table 4:** Plant hormones remain stable 48 h after extraction.

Analyte/IS	RRT^a^ RDS (%)	RPA^b^ RDS (%)
**Z/d_5_-Z**	0.76	9.47
**IAA/d_5_-IAA**	0.43	8.96
**ABA/d_6_-ABA**	0.25	5.05
**JA/d_5_-JA**	0.26	1.72
**SA/d_4_-SA**	0.27	2.79
**ACC/d_4_-ACC**	1.35	7.32
**GA_4_/d_2_-GA_4_**	0.24	7.11
**GA_9_/d_2_-GA_9_**	0.18	5.17
**GA_24_/d_2_-GA_24_**	0.23	2.56

^a ^Relative retention time

^b ^Relative peak area

### Hormonal profiling of rosemary leaves under salt stress

**Figure 3:**
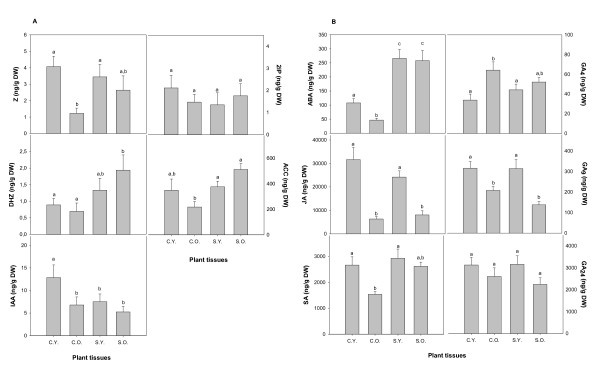
**Salt stress alters the hormonal balance of leaves**. Concentration of endogenous plant hormones (A) Z, DHZ, 2iP, IAA, ACC, and (B) ABA, JA, SA, GA_4_, GA_9_, GA_24 _found in rosemary leaves compared to control and salt-stressed plants (200 mM NaCl for 8 days). C.Y., young leaves of control plants; C.O., old leaves of control plants; S.Y., young leaves of salt-stressed plants; S.O., old leaves of salt-stressed plants.

Rosemary is a moderately salt-tolerant glycophyte [28,29]. Plants exposed to 200 mM NaCl during the experiment suffered salt stress indicated by a decrease of RWC and the *F*_v_/*F*_m _ratio (Table 5). It has been shown that a number of plant hormones play pivotal roles to overcome damaging effects of salinity in plants, mainly the anti-stress defense compounds ABA, ethylene, SA and JA [24]. ABA regulates not only stomatal closure and hydraulic conductivity, but also root and shoot growth under salt stress conditions [30,31]. Ethylene has also been reported to be involved in salt-induced senescence [32,33]. It has been found increasing amounts of ACC, the precursor of ethylene, in tomato plants during salinity [22]. SA and JA have also been suggested to be involved in cellular signaling in plant response to salinity [34-36]. Furthermore, essential regulators of plant growth, such as cytokinins, auxins and GAs are also sensitive to experience changes during salt stress responses, since this environmental constraint is known to inhibit leaf growth. Therefore, obtaining a hormone profile is essential to understand the delicate balance of regulators in plant responses to salinity, and the profiling obtained will obviously depend on the species examined, duration and magnitude of stress and specific conditions of study. Here we present the hormone profile of rosemary plants in response to salinity with a distinction in the response between young and old leaves (Figure 3). Results show that senescence in old leaves of control plants is associated with a 75% reduction of the active cytokinin, zeatin (while other cytokinins are not significantly altered) and 40% or even higher reductions in ABA, JA and SA. It was found that this hormone profile (differences between old and young leaves) is completely altered in salt-stressed plants, in which neither cytokinin nor the anti-stress defense compounds ABA and SA decrease significantly in old leaves, however, JA levels remain constant. In other words, old leaves of salt-stressed plants showed higher levels of Z, ABA, and SA than old leaves of control plants, although differences were significant for ABA only, with 8-fold higher levels in old leaves of salt-stressed compared to control plants. It appears from this hormone profile that reductions in Z levels may trigger leaf senescence in control plants, increases in ABA are the responsible for triggering plant defense in both young and old leaves of salt-stressed plants. Obviously, this response will be affected by a cross-talk between different signaling pathways that need to be evaluated in depth using other complementary experimental approaches.

**Table 5:** Relative water content (RWC) and maximum efficiency of PSII photochemistry (F_v_/F_m _ratio, indicative of damage to PSII) in *Rosmarinus officinalis *leaves of control and salt-stressed plants (treated with 200 mM NaCl for 8 days).

	RWC (%)	F_v_/F_m_
**Control young leaves**	80.83 ± 1.63^a^	0.837 ± 0.02^a^
**Control old leaves**	83.09 ± 1.57^a^	0.837 ± 0.01^a^
**Stress young leaves**	73.83 ± 1.78^b^	0.728 ± 0.03^b^
**Stress old leaves**	61.32 ± 1.10^c^	0.633 ± 0.04^b^

Special mention deserves the case of GAs, since more than 100 GAs are found in plants, although only a few of these are known to have biological activity such as GA_4 _[37]. Of the six analyzed gibberellins, only GA_4_, GA_9 _and GA_24 _were found in rosemary leaves. GA_4 _levels showed significant higher levels about 40% for old leaves in control plants compared to young leaves (Figure 3). Interestingly, significant higher levels of GA_9_, the immediate precursor of GA_4_, were observed for young leaves of both control and salt-stressed plants compared to old leaves, thus indicating a specific GA_9 _to GA_4 _conversion with the induction of leaf senescence in rosemary plants. However, it should be noted that GA_4 _levels were an order of magnitude lower than those of GA_9_, thus suggesting that the latter is precursor of different GAs.

To our knowledge we present the first hormone profile including ABA, ACC, auxins, cytokinins, GAs, JA and SA of rosemary plants in response to salinity comparing young and old leaves. In the literature ABA, IAA, ACC and cytokinin levels have been reported to be altered in *R. officinalis *leaves under water stress using ELISA and GC-MS/MS analyses (Table 6) [38,39]. While ABA and ACC levels were comparable to our results, auxin levels were 20-fold higher than those obtained here. Furthermore, ZR was reported to occur in this species, while this cytokinin was not detected in the present study. In this previous study, the cross-reactivity of the antibodies used was as follows: ZR at 100% and Z at 88%, which could explain at least partly the discrepancy between both studies. Aside from the differences in the experimental approaches used, plant and leaf developmental stages, life history traits, plant varieties and type, duration and magnitude of stress imposed on plants will also determine the differences in the levels of the different hormones. It is shown here, for instance, how salt stress and leaf age can significantly alter the hormone profile of leaves.

**Table 6:** Comparing plant hormones contents from *Rosmarinus officinalis *leaves analyzed by different methods.

	ELISA [38]	GC-MS/MS [38,39]	UPLC-MS/MS (this study)
	(pmol/g FW)	(pmol/g DW)	(pmol/g DW)
**ABA**	-	400-1500	200-1000
**ACC**	-	1000-16000	2000-5000
**IAA**		200-1500	30-70
**ZR equiv**.	10000-30000	-	-
**Experimental conditions**	Water-stressed plants under controlled conditions	Field-grown plants exposed to summer drought	Salt-stressed plants under controlled conditions

## Conclusions

**Figure 4:**
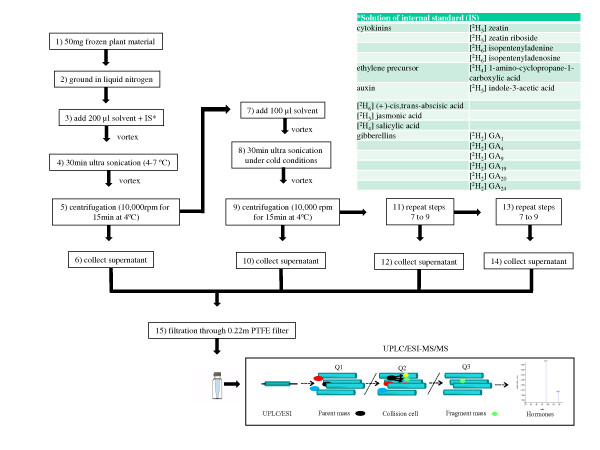
**Summary of the extraction protocol and analysis**. Schematic extraction diagram to analyze small amounts of plant material using UPLC/ESI-MS/MS with multiple reaction monitoring (MRM).

The development of a rapid, sensitive, high throughput, cost effective method for quantification of 17 endogenous plant hormones from seven plant classes in a complex matrix is described. The use of UPLC/ESI-MS/MS with multiple reaction monitoring (MRM) allowed each sample could be analyzed within 6 minutes. The method requires minimal plant tissue, is highly reproducible and was applicable to analyze dynamic changes in endogenous concentrations of hormones in plants exposed to salt stress. Due to the structural diversity of plant hormones no one extraction solvent was capable of extract all plant hormones equally well. If we aim at reducing extraction steps to a minimum, depending on the plant hormones of interest a different solvent is recommended: methanol:isopropanol:glacial acetic acid, 20:79:1 (v/v/v) for ABA, SA, JA, IAA and GAs, methanol:isopropanol:glacial acetic acid, 60:39:1 (v/v/v) for cytokinins, and methanol:glacial acetic acid, 99:1 (v/v) for ACC. Furthermore, this method is equally applicable to fresh or freeze dried tissue. Moreover, high loss of plant hormones during drying of sample extracts under N_2 _and re-suspension of the residues indicates that sample extracts should immediately be injected. However, the adjustment of sample weight and volume of the extraction solvent must be taken into account. As a summary, a schematic extraction diagram to analyze small amounts of plant material without time-consuming additional steps such as purification, sample drying under N_2 _and re-suspension of the residues using UPLC/ESI-MS/MS with MRM is shown in Figure 4. This method has been used in our group to obtain the hormone profile of a number of plant tissues, including leaves, flowers and seeds of different species of a number of plant families (including the model plant *Arabidopsis thaliana *and other Brassicaceae, Liliaceae, Cistaceae, Anacardiaceae, Dioscoreaceae, Velloziaceae, Xyridaceae and Lamiaceae) that all bring completely different matrices and therefore their specific complexity for analyses. As shown in the present study, each species will require a specific handling during extraction and analysis, and the schematic diagram presented in Figure 4 can serve as a basis to the optimization of the method for each species. Although obtaining the hormone profile of species with complex matrices may be more difficult due to the presence of possible interfering compounds, it is also challenging and will undoubtedly be needed in the near future if we are to better understand plant stress responses in species of contrasting habitats that can be used as model plants for the study of plant responses to environmental stresses.

## Methods

### Chemicals

Unlabeled ACC, IPA, Z, ZR, IAA, ABA, JA, SA were purchased from Sigma-Aldrich (Steinheim, Germany). Unlabeled 2iP, DHZ, DHZR, and deuterium labeled d_4_-ACC, d_6_-IPA, d_5_-Z, d_5_-ZR, d_5_-IAA, d_6_-ABA, d_4_-SA, d_5_-JA and d_6_-2iP were purchased from OlChemim Ltd. (Olomouc, Czech Republic). Unlabeled and deuterium labeled gibberellins GA_1_, GA_4_, GA_9_, GA_19_, GA_20_, GA_24_, d_2_-GA_1_, d_2_-GA_4_, d_2_-GA_9_, d_2_-GA_19_, d_2_-GA_20_, d_2_-GA_24 _were purchased from Dr. Lewis Mander at the Australian National University (Canberra, Australia).

### Plant material and sampling

All work was carried out with rosemary leaves. Fifteen plants were purchased from a nursery (Vic, Spain) and maintained in a greenhouse at the experimental fields of the University of Barcelona (Barcelona, Spain) with controlled temperature (24/18°C) and adequate water conditions by irrigating the plants with half concentration of Hoagland solution every 2 days. For the optimization procedure of sample preparation and extraction, leaves from these plants were collected, frozen in liquid nitrogen and stored at -80°C until analysis. For the salinity experiment, five plants were exposed to salt stress by irrigating with the same nutrient solution containing an extra addition of 200 mM NaCl, and compared to five control plants (all plants were watered every 2 days). Young and old leaves from the uppermost and lowest part of the plant, respectively, were collected after 8 days of treatment, frozen in liquid nitrogen and stored at -80°C until analyses.

### Leaf water content and chlorophyll fluorescence

To determine the relative water content (RWC) of leaf material from the salinity experiment, young and old leaves were collected, immediately weighed (FW), re-hydrated for 24 h at 4°C in darkness (TW) and subsequently oven-dried for 48 h at 60°C (DW). The RWC was determined as 100 × (FW-DW)/(TW-DW). Measurements of the maximum efficiency of photosystem II photochemistry (F_v_/F_m _ratio) were made by using a pulse-modulated fluorimeter Imaging-PAM (Walz, Effeltrich, Germany) after 2 h of dark adaptation. The *F_v_/F_m _*ratio was calculated as (F_m_-F_0_)/Fm, where F_m _and F_0 _are the maximum and basal fluorescence yields, respectively, of dark-adapted leaves.

### Sample preparation

Frozen leaf material (about 100 mg FW.) was ground in liquid nitrogen with the mixer mill MM400 (Retsch GmbH, Haan, Germany) in a 2 ml Eppendorf tube, and then extracted with 1 ml of extraction solvent (methanol:isopropanol, 20:80 (v/v) with 1% of glacial acetic acid) using ultra sonication (4-7°C). The labeled forms of the compounds d_4_-SA, d_6_-ABA, d_5_-JA, d_5_-IAA, d_2_-GA_1_, d_2_-GA_4_, d_2_-GA_9_, d_2_-GA_19_, d_2_-GA_20_, d_2_-GA_24_, d_4_-ACC, d_6_-2iP, d_6_-IPA, d_5_-Z and d_5_-ZR were added as internal standards. D_5_-Z and d_5_-ZR were used as internal standards for DHZ and DHZR, respectively. After centrifugation (10,000 rpm for 15 min at 4°C), the supernatant was collected and the pellet was re-extracted with 0.5 ml of extraction solvent and the extraction repeated three times again. Then, supernatants were combined and dried completely under a nitrogen stream and re-dissolved in 300 μl of methanol, centrifuged (10,000 rpm for 5 min) and filtered through a 0.22 μm PTFE filter (Waters, Milford, MA, USA). Samples (5 μl) were then analyzed by UPLC/ESI-MS/MS. Hormones were determined in ten independent samples for each treatment. Quantification was done by the creation of calibration curves including each of the 17 unlabeled analyte compounds (SA, ABA, JA, IAA, GA1, GA_4_, GA_9_, GA_19_, GA_20_, GA_24_, ACC, 2iP, IPA, Z, ZR, DHZ and DHZR). Ten standard solutions were prepared ranging from 0.05 to 1000 ng ml^-1 ^and for each solution a constant amount of internal standard (as described above) was added. Calibration curves for each analyte were generated using Analyst™ software (Applied Biosystems, Inc., California, USA). The limit of detection (LOD, S/N = 3) and the limit of quantification (LOQ, S/N = 10) were also calculated with the aid of this software.

### UPLC/ESI-qMS/MS analysis

The UPLC system consisted of an Aquity UPLC™ System (Waters, Milford, MA USA) quaternary pump equipped with an autosampler. For the analysis of the extracts, a HALO™ C18 (Advanced Materials Technology, Inc., Wilmington, USA) column (2.1 × 75 mm, 2.7 μm) was used. Gradient elution was done with water and 0.05% glacial acetic acid (solvent A) and acetonitrile with 0.05% glacial acetic acid (solvent B) at a constant flow rate of 0.6 ml min^-1^. Cytokinins and ACC were analyzed using method 1 (M1) and ABA, JA, SA, IAA, and gibberellins were analyzed using method 2 (M2). The gradient profile for M1 (cytokinins and ACC) was applied as follow (*t *(min), % A): (0, 99), (2, 0), (2.40, 0), (2.60, 99), (3, 99). The gradient profile for M2 (ABA, JA, SA, IAA, and gibberellins) was applied as follow: (*t *(min), % A): (0, 99), (2.20, 0), (2.40, 0), (2.60, 99), (3, 99). MS and MS/MS experiments were performed on an API 3000 triple quadrupole mass spectrometer (PE Sciex, Concord, Ont., Canada). Analyses for M1 were performed using Turbo Ionspray source in positive ion mode and for M2 in negative ion mode. For both methods temperature was 400°C, nebulizer gas (N_2_) 10 (arbitrary units), curtain gas (N_2_) 12 (arbitrary units), collision gas (N_2_) 4 (arbitrary units) and the capillary voltage was 3.5 kV for M1 and -3.5 kV for M2, respectively. The optimized MS/MS conditions for the analysis of plant hormones are summarized in Additional File 4 and were determined in infusion experiments: a standard solution of each plant hormone and deuterium labeled plant hormone was infused of a constant flow rate of 15 μl min^-1 ^into the mass spectrometer using a Model 11 syringe pump (Harvard Apparatus, Holliston, MA, USA). The mass spectrometer was operated in multiple reaction mode (MRM) due to their high selectivity using precursor-to-product ion transitions because many compounds could present the same nominal molecular mass or peaks can overlap. Since more than 100 GAs with partly same molecular masses and similar retention times are found in plants special mention is needed for GAs identification in plant extracts. Additional files 5 and 6 show fragmentation patterns for labeled and unlabeled GA_1_, GA_4_, GA_9_, GA_19_, GA_20_, GA_24 _standards. In rosemary leaf extracts GA_4_, GA_9 _and GA_24 _were detected. Using multiple reaction monitoring (MRM) conditions a specific precursor to one product ion transition is monitored. However, to verify the identification of GAs in rosemary leaf extracts the specific precursor ions of GA_4_, GA_9 _and GA_24 _to two different product ions in MRM mode were monitored. All GAs mass chromatograms from rosemary leaf extracts showed identical retention times as GA standards.

### Statistical analyses

Differences between treatments were evaluated using the analysis of variance (ANOVA), using the DMS's post hoc test, and were considered significant at a probability level of P < 0.05.

## Abbreviations

ABA: abscisic acid; ACC: 1-amino-cyclopropane-1-carboxyic acid; CE: collision energy: CXP: collision cell exit potential; DHZ: dihydrozeatin; DHZR: dihydrozeatin riboside; DP: declustering potencial; EP: entrance potential; ESI-MS/MS: electrospray ionization tandem mass spectrometry; FP: focusing potential; GA: gibberellin; IAA: índole-3-acetic acid; 2iP: isopentenyladenine: IPA: isopentenyladenosine; JA: jasmonic acid; LOD: limit of detection; LOQ: limit of quantification; MRM: multiple reaction monitoring; OPDA: 12-oxo-phytodienoic acid; RDS: relative deviation standard; SA: salicylic acid; UPLC: ultrahigh performance liquid chromatography; Z: zeatin; ZR: zeatin riboside.

## Competing interests

The authors declare that they have no competing interests.

## Authors' contributions

MM participated in the design of the study, carried out the lab work and helped to draft the manuscript. SMB conceived of the study, and participated in its design and coordination and helped to draft the manuscript. All authors have read and approved the final manuscript.

## Supplementary Material

Additional file 1 **Effects of freeze-drying**. Concentration of endogenous plant hormones (A) Z, DHZ, 2iP, IAA, ACC, and (B) ABA, JA, SA, GA_4_, GA_9_, GA_24 _detected in fresh weight (FW) and freeze-dried (DW) rosemary leaves. Click here for file

Additional file 2 **Extraction efficiency**. Concentration of (A) Z, DHZ, 2iP, IAA, ACC, and (B) ABA, JA, SA, GA_4_, GA_9_, GA_24 _found in rosemary leaves after 1, 2, 3, 4 and 5 extraction procedures. Click here for file

Additional file 3 **Effects of the amount of plant material used for extraction**. Capacity of extraction method to analyze different leaf amounts (20 - 200 mg FW) of rosemary leaves. Click here for file

Additional file 4 **Optimized UPLC/ESI-MS/MS parameters**. Parameters are listed in multiple reaction monitoring (MRM) conditions for quantification of plant hormones. Click here for file

Additional file 5 **Fragmentation patterns of labeled and unlabeled GA_1_, GA_19_, and GA_20 _standards**. (A) GA_1 _(precursor m/z 347 and product m/z 273 ions) and d_2_-GA_1 _standards (precursor m/z 349 and product m/z 275 ions). (B) GA_19 _(precursor m/z 361 and product m/z 273 ions) and d_2_-GA_19 _standards (precursor m/z 363 and product m/z 275 ions), (C) GA_20 _(precursor m/z 331 and product m/z 287 ions) and d_2_-GA_20 _standards (precursor m/z 333 and product m/z 289 ions) Click here for file

Additional file 6 **Fragmentation patterns of labeled and unlabeled GA_4_, GA_9_, GA_24 _standards and identification of GA_4_, GA_9 _and GA_24 _in *Rosmarinus officinalis *extracts**. (A1) Fragmentation patterns of GA_4 _(precursor m/z 331 and product m/z 213 ions) and d_2_-GA_4 _standards (precursor m/z 333 and product m/z 215 ions). (A2) UPLC/ESI-MS/MS chromatograms from rosemary leaf extract using multiple reaction monitoring (MRM) conditions. GA_4 _was identified by monitoring the precursor ion (*m/z *331) to two different product ion transitions (*m/z *213 and *m/z *225) in MRM mode with identical retention times as the GA_4 _standard. (B1) Fragmentation patterns of GA_9 _(precursor m/z 315 and product m/z 271 ions) and d_2_-GA_9 _standards (precursor m/z 317 and product m/z 273 ions). (B2) UPLC/ESI-MS/MS chromatograms from rosemary leaf extract using multiple reaction monitoring (MRM) conditions. GA_9 _was identified by monitoring the precursor ion (*m/z *315) to two different product ion transitions (*m/z *271 and *m/z *253) in MRM mode with identical retention times as the GA_9 _standard. (C1) Fragmentation patterns of GA_24 _(precursor m/z 345 and product m/z 257 ions) and d_2_-GA_24 _standards (precursor m/z 347 and product m/z 259 ions). (C2) UPLC/ESI-MS/MS chromatograms from rosemary leaf extract using multiple reaction monitoring (MRM) conditions. GA_24 _was identified by monitoring the precursor ion (*m/z *345) to two different product ion transitions (*m/z *257 and *m/z *301) in MRM mode with identical retention times as the GA_24 _standard. Click here for file
