# Supplementary figures and images for: Rapid and sensitive hormonal profiling of complex plant samples by liquid chromatography coupled to electrospray ionization tandem mass spectrometry
Source: Plant Methods. 2011 Nov 18;7:37. doi: 10.1186/1746-4811-7-37 (PMC3253682; doi:10.1186/1746-4811-7-37)

## Slide 1
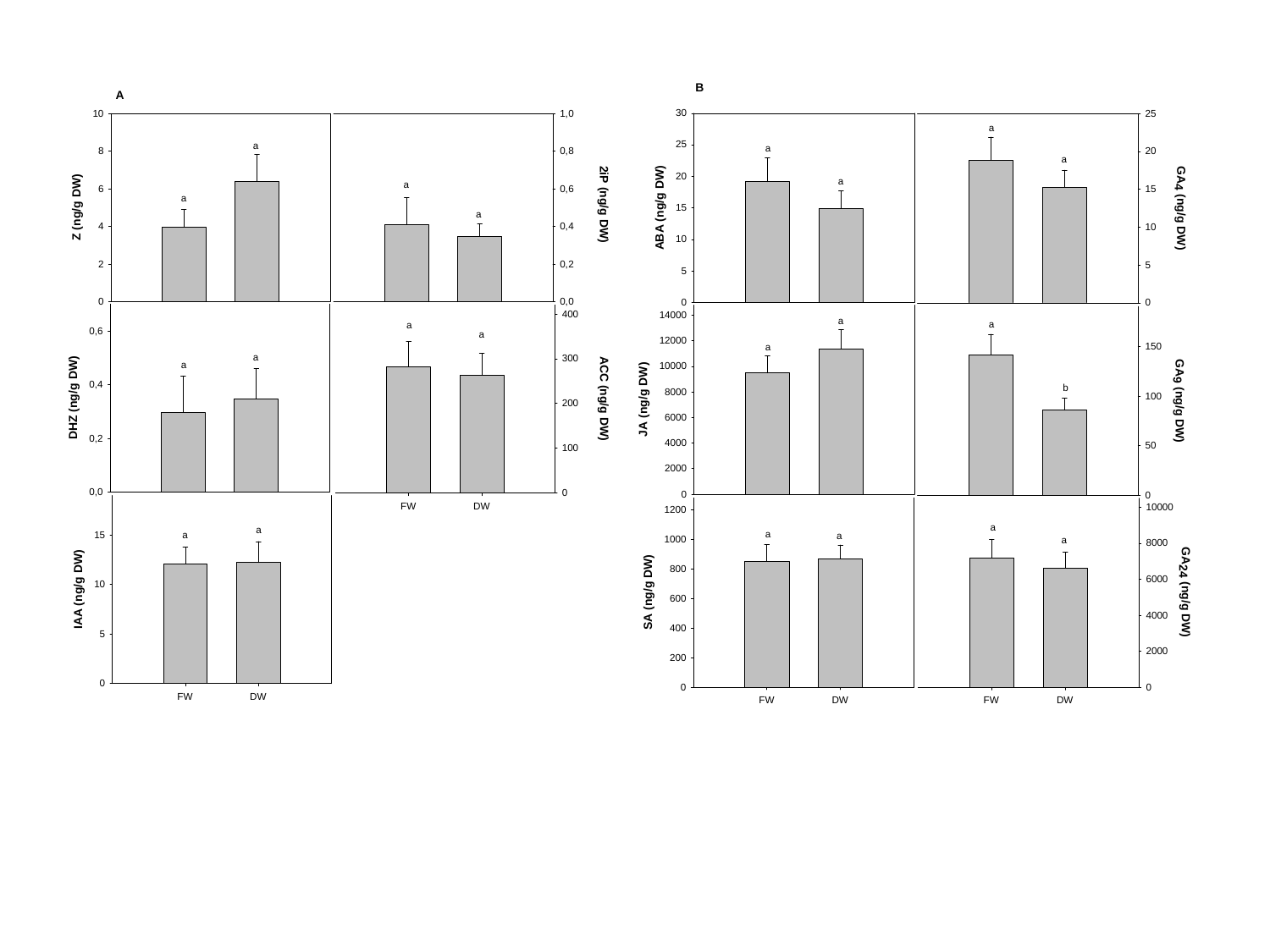

Supplement: Additional file 1 — Effects of freeze-drying. Concentration of endogenous plant hormones (A) Z, DHZ, 2iP, IAA, ACC, and (B) ABA, JA, SA, GA4, GA9, GA24 detected in fresh weight (FW) and freeze-dried (DW) rosemary leaves. [file 1746-4811-7-37-S1.PPT]

## Slide 1
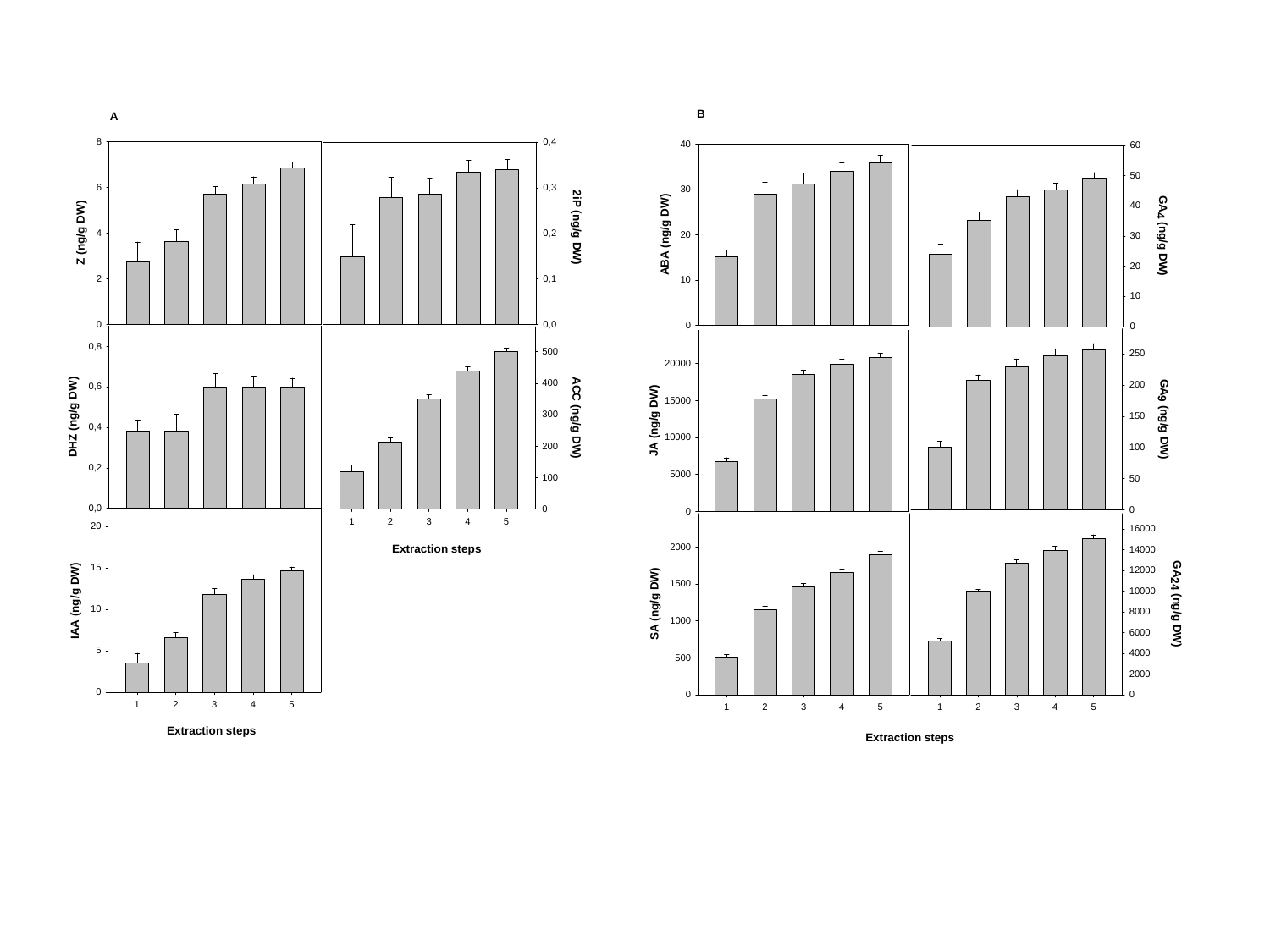

Supplement: Additional file 2 — Extraction efficiency. Concentration of (A) Z, DHZ, 2iP, IAA, ACC, and (B) ABA, JA, SA, GA4, GA9, GA24 found in rosemary leaves after 1, 2, 3, 4 and 5 extraction procedures. [file 1746-4811-7-37-S2.PPT]

## Slide 1
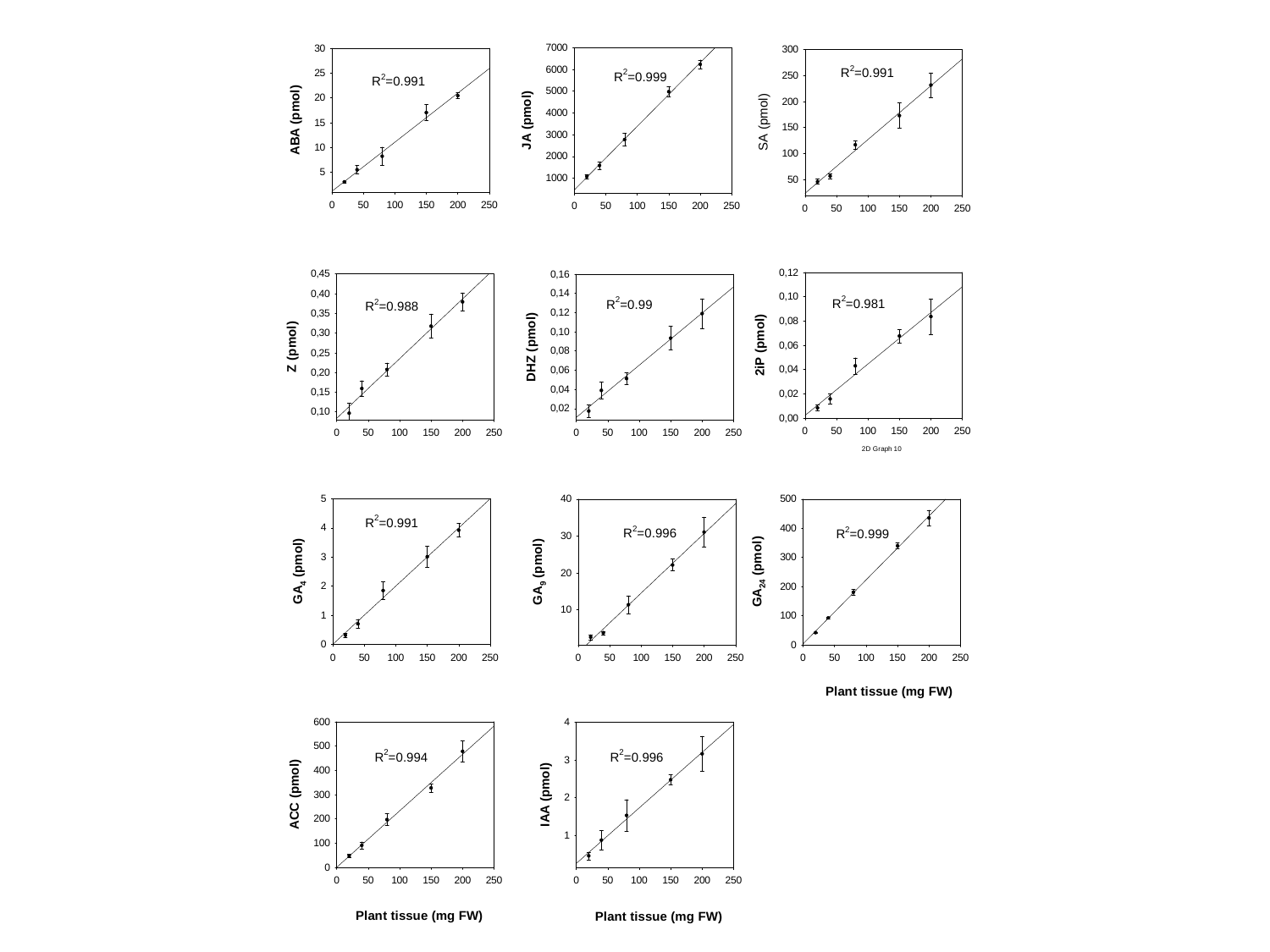

Supplement: Additional file 3 — Effects of the amount of plant material used for extraction. Capacity of extraction method to analyze different leaf amounts (20 - 200 mg FW) of rosemary leaves. [file 1746-4811-7-37-S3.PPT]
