# Supplementary material for: Rapid and sensitive hormonal profiling of complex plant samples by liquid chromatography coupled to electrospray ionization tandem mass spectrometry
Source: Plant Methods. 2011 Nov 18;7:37. doi: 10.1186/1746-4811-7-37 (PMC3253682; doi:10.1186/1746-4811-7-37)
Supplement: Additional file 4 — Optimized UPLC/ESI-MS/MS parameters. Parameters are listed in multiple reaction monitoring (MRM) conditions for quantification of plant hormones. [file 1746-4811-7-37-S4.RTF]

Additional File 4. Optimized UPLC/ESI-MS/MS parameters listed in multiple reaction mode (MRM) for quantification of plant hormones.
Analyte and IS	Retention time
(min)	Transition	Scan mode	DP 
	FP
	EP
	CE
	CXP
	
M1								
ACC	0.28	102.1 > 56.2	+	30	200	10	15	15	
d4-ACC	0.28	106 > 60.1	+	30	200	10	15	15	
t-Z	0.68	220 > 136	+	40	200	10	25	15	
d5-t-Z	0.68	225 > 137	+	40	200	10	25	15	
DHZ	0.69	222 > 136	+	50	200	10	40	10	
t-ZR	0.75	352 > 220	+	45	200	10	25	15	
d5-t-ZR	0.75	357 > 225	+	45	200	10	25	15	
DHZR	0.76	354 > 222.2	+	55	200	10	40	10	
2iP	0.95	204 > 136.1	+	50	200	5	35	10	
d6-2iP	0.95	210 > 137.2	+	50	190	5	35	10	
IPA	1.01	336 > 204	+	45	200	10	25	15	
d6-IPA	1.00	342 > 210.2	+	45	200	10	25	15	
										
M2								
GA1	0.94	347 > 273	-	-65	-200	-10	-40	-15	
d2-GA1	0.94	349 > 275	-	-65	-200	-9	-40	-15	
SA	1.04	137 > 93	-	-30	-200	-10	-20	-15	
d4-SA	1.04	141 > 97.2	-	-30	-200	-10	-20	-15	
GA19	1.11	361 > 273.5	-	-60	-225	-12.5	-50	-10	
d2-GA19	1.10	363 > 275	-	-90	-225	-12.5	-50	-10	
IAA	1.12	174 > 130	-	-35	-200	-10	-15	-15	
d5-IAA	1.11	179 > 135	-	-35	-200	-10	-25	-15	
ABA	1.16	263 > 154	-	-55	-200	-10	-30	-15	
d6-ABA	1.15	269 > 159	-	-55	-200	-10	-30	-15	
JA	1.31	209 > 59	-	-37	-200	-10	-25	-15	
d5-JA	1.31	214 > 64	-	-37	-200	-10	-25	-15	
GA20	1.31	331 > 287	-	-70	-230	-10	-40	-15	
d2-GA20	1.31	333 > 289.3	-	-70	-230	-10	-40	-15	
GA4	1.40	331 > 213	-	-50	-200	-10	-40	-15	
d2-GA4	1.39	333 > 215	-	-50	-200	-10	-40	-15	
GA24	1.45	345 > 257	-	-70	-200	-10	-50	-15	
d2-GA24	1.44	347 > 259	-	-75	-200	-10	-50	-15	
GA9	1.64	315 > 271.3	-	-90	-225	-12.5	-50	-10	
d2-GA9	1.63	317 > 273	-	-60	-140	-9	-40	-15	
ACC, 1-amino-cyclopropane-1-carboxyic acid; Z, zeatin, DHZ, dihydrozeatin; ZR,  zeatin riboside, DHZR, dihydrozeatin riboside; K, kinetin; 2iP,  isopentenyladenine, IPA, isopentenyladenosine; GA, gibberellins; SA, salicylic acid;  IAA, índole-3-acetic acid, ABA, abscisic acid, JA, jasmonic acid; DP, declustering potencial; FP, focusing potencial; EP, entrance potential; CE, collision energy; CXP, collision cell exit potencial.
