# Supplementary material for: Rapid and sensitive hormonal profiling of complex plant samples by liquid chromatography coupled to electrospray ionization tandem mass spectrometry
Source: Plant Methods. 2011 Nov 18;7:37. doi: 10.1186/1746-4811-7-37 (PMC3253682; doi:10.1186/1746-4811-7-37)
Supplement: Additional file 5 — Fragmentation patterns of labeled and unlabeled GA1, GA19, and GA20 standards. (A) GA1 (precursor m/z 347 and product m/z 273 ions) and d2-GA1 standards (precursor m/z 349 and product m/z 275 ions). (B) GA19 (precursor m/z 361 and product m/z 273 ions) and d2-GA19 standards (precursor m/z 363 and product m/z 275 ions), (C) GA20 (precursor m/z 331 and product m/z 287 ions) and d2-GA20 standards (precursor m/z 333 and product m/z 289 ions) [file 1746-4811-7-37-S5.PPT]

## Slide 1
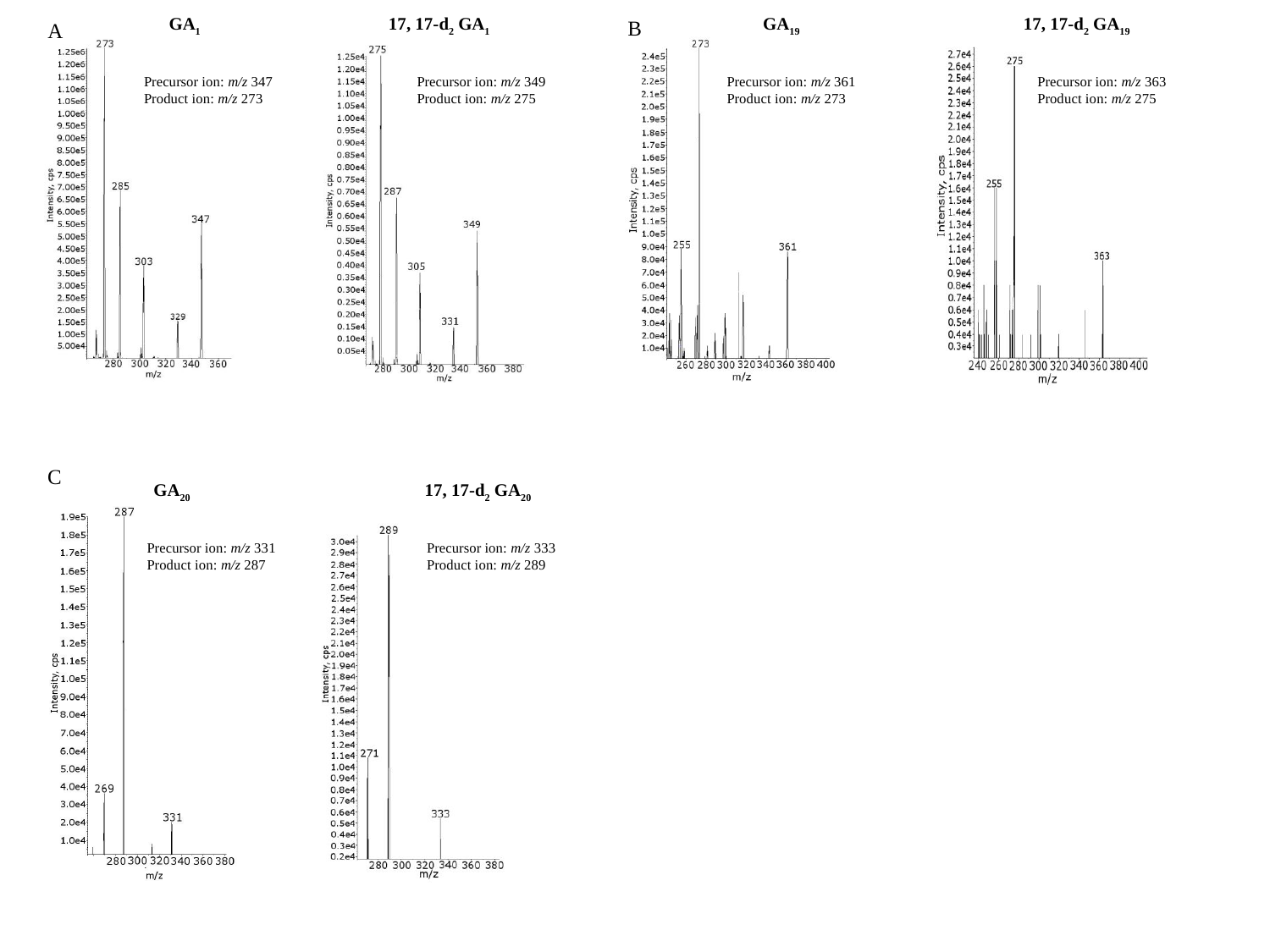

GA1
17, 17-d2 GA1
GA19
 17, 17-d2 GA19
B
A
Precursor ion: m/z 347
Product ion: m/z 273
Precursor ion: m/z 349
Product ion: m/z 275
Precursor ion: m/z 361
Product ion: m/z 273
Precursor ion: m/z 363
Product ion: m/z 275
C
 GA20
 17, 17-d2 GA20
Precursor ion: m/z 331
Product ion: m/z 287
Precursor ion: m/z 333
Product ion: m/z 289
