# Supplementary material for: Rapid and sensitive hormonal profiling of complex plant samples by liquid chromatography coupled to electrospray ionization tandem mass spectrometry
Source: Plant Methods. 2011 Nov 18;7:37. doi: 10.1186/1746-4811-7-37 (PMC3253682; doi:10.1186/1746-4811-7-37)
Supplement: Additional file 6 — Fragmentation patterns of labeled and unlabeled GA4, GA9, GA24 standards and identification of GA4, GA9 and GA24 in Rosmarinus officinalis extracts. (A1) Fragmentation patterns of GA4 (precursor m/z 331 and product m/z 213 ions) and d2-GA4 standards (precursor m/z 333 and product m/z 215 ions). (A2) UPLC/ESI-MS/MS chromatograms from rosemary leaf extract using multiple reaction monitoring (MRM) conditions. GA4 was identified by monitoring the precursor ion (m/z 331) to two different product ion transitions (m/z 213 and m/z 225) in MRM mode with identical retention times as the GA4 standard. (B1) Fragmentation patterns of GA9 (precursor m/z 315 and product m/z 271 ions) and d2-GA9 standards (precursor m/z 317 and product m/z 273 ions). (B2) UPLC/ESI-MS/MS chromatograms from rosemary leaf extract using multiple reaction monitoring (MRM) conditions. GA9 was identified by monitoring the precursor ion (m/z 315) to two different product ion transitions (m/z 271 and m/z 253) in MRM mode with identical retention times as the GA9 standard. (C1) Fragmentation patterns of GA24 (precursor m/z 345 and product m/z 257 ions) and d2-GA24 standards (precursor m/z 347 and product m/z 259 ions). (C2) UPLC/ESI-MS/MS chromatograms from rosemary leaf extract using multiple reaction monitoring (MRM) conditions. GA24 was identified by monitoring the precursor ion (m/z 345) to two different product ion transitions (m/z 257 and m/z 301) in MRM mode with identical retention times as the GA24 standard. [file 1746-4811-7-37-S6.PPT]

## Slide 1
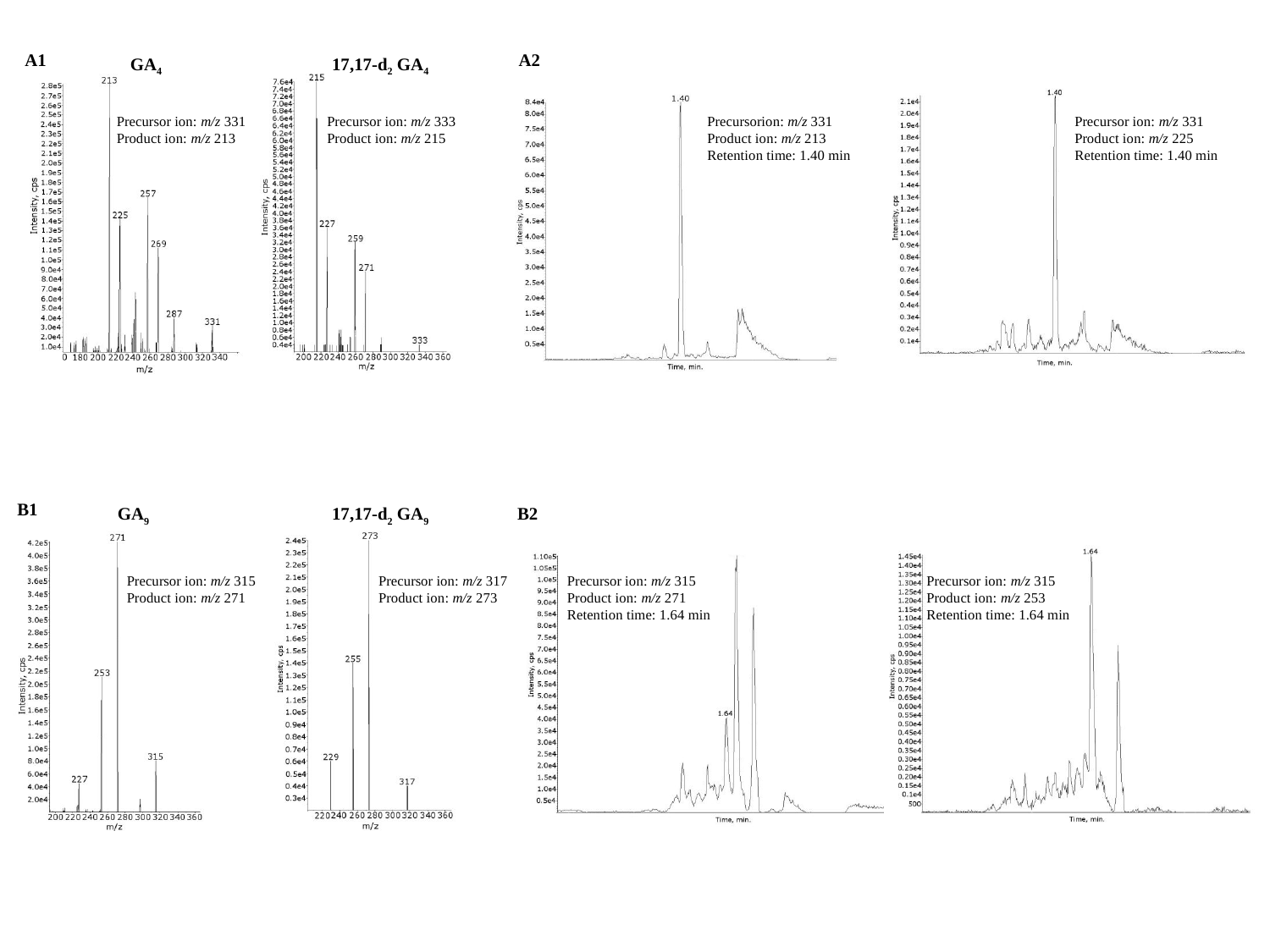

A1
A2
GA4
 17,17-d2 GA4
Precursor ion: m/z 331
Product ion: m/z 213
Precursor ion: m/z 333
Product ion: m/z 215
Precursorion: m/z 331
Product ion: m/z 213
Retention time: 1.40 min
Precursor ion: m/z 331
Product ion: m/z 225
Retention time: 1.40 min
B1
GA9
 17,17-d2 GA9
B2
Precursor ion: m/z 315
Product ion: m/z 271
Precursor ion: m/z 317
Product ion: m/z 273
Precursor ion: m/z 315
Product ion: m/z 271
Retention time: 1.64 min
Precursor ion: m/z 315
Product ion: m/z 253
Retention time: 1.64 min

## Slide 2
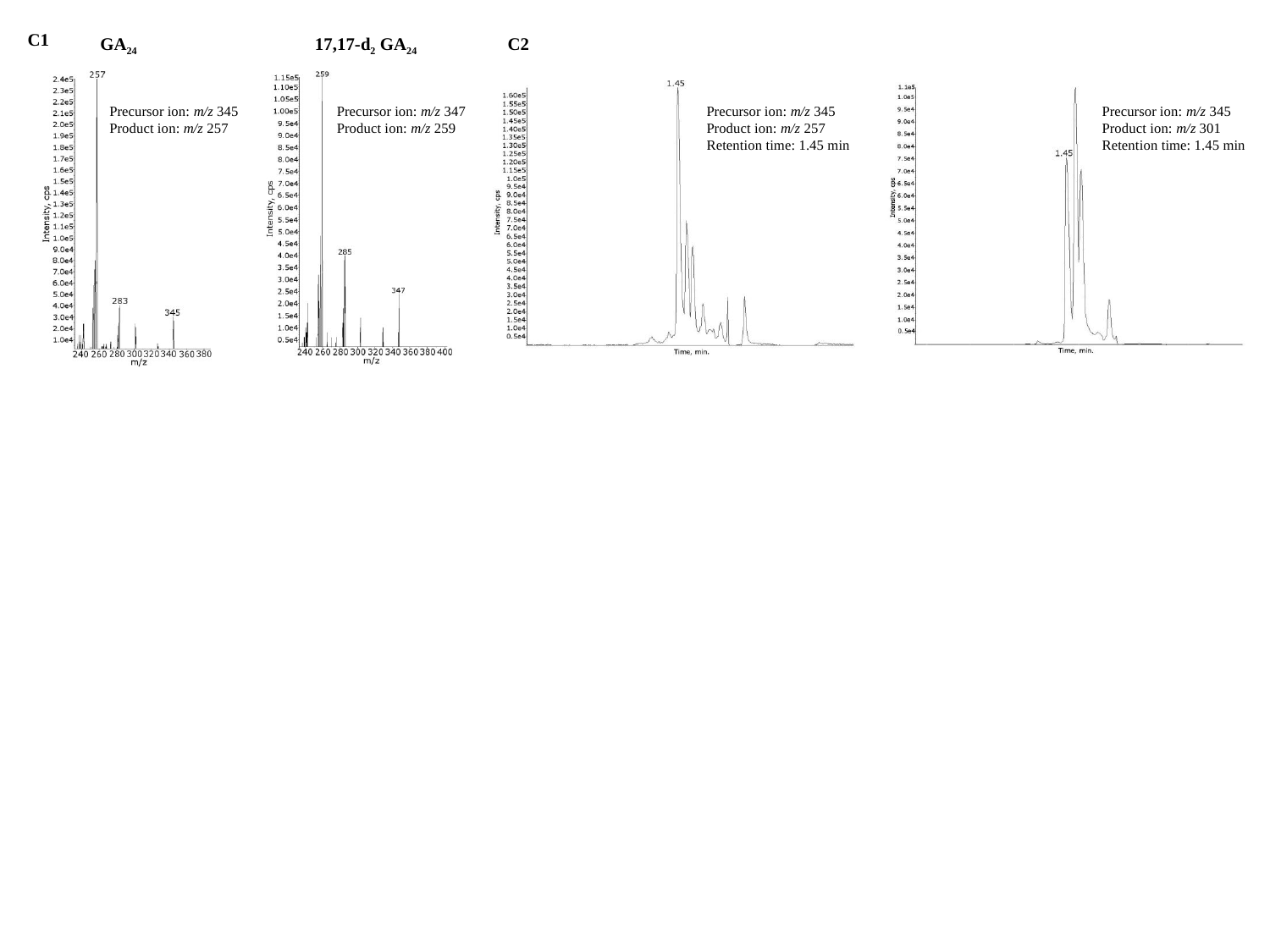

C1
GA24
 17,17-d2 GA24
C2
Precursor ion: m/z 345
Product ion: m/z 257
Precursor ion: m/z 347
Product ion: m/z 259
Precursor ion: m/z 345
Product ion: m/z 257
Retention time: 1.45 min
Precursor ion: m/z 345
Product ion: m/z 301
Retention time: 1.45 min
